# Monitoring of Pore Orientation by *in Operando* Grazing Incidence Small-Angle X-ray Scattering during Templated Electrodeposition of Mesoporous Pt Films

**DOI:** 10.1021/acsami.3c03316

**Published:** 2023-09-28

**Authors:** Philipp Aldo Wieser, David Moser, Bernhard Gollas, Heinz Amenitsch

**Affiliations:** † Institute of Inorganic Chemistry, Graz University of Technology, Graz 8010, Austria; ‡ Institute of Electron Microscopy and Nanoanalysis, Graz University of Technology, Graz 8010, Austria; § Institute for Chemistry and Technology of Materials, Graz University of Technology, Graz 8010, Austria

**Keywords:** templated electrochemical deposition, *in operando* GISAXS, mesoporous platinum films

## Abstract

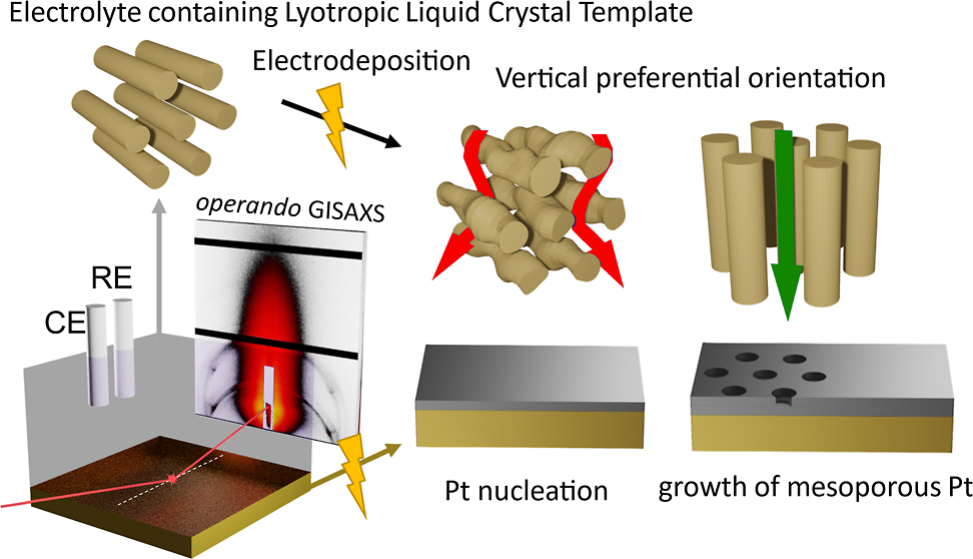

We have used *in operando* grazing incidence small-angle X-ray scattering (GISAXS) to monitor structural changes during templated electrodeposition of mesoporous platinum films on gold electrodes from a ternary lyotropic liquid crystalline mixture of aqueous hexachloroplatinic acid and the diblock copolymer surfactant Brij56. While the cylindrical micelles of the lyotropic liquid crystal (LLC) in the hexagonal phase have a center-to-center distance of 7.5 nm with a preferential alignment parallel to the electrode surface, the electrodeposited platinum films contain highly ordered mesopores arranged in a 2D hexagonal structure, with a center-to-center distance of about 8.5 nm and a preferential orientation perpendicular to the electrode surface. The progression of structural changes of the LLC template and the deposited mesoporous Pt could be monitored for the first time *in operando* by GISAXS: within the first 14 s of deposition, a nucleation burst of Pt coincides with a loss of preferential alignment of the LLC. Initially, the morphology of the 2-dimensionally nucleated Pt replicates the Au substrate. During the following 5 to 7 min, the growth morphology of the Pt film changes, and vertically aligned mesopores form. Our results indicate mutual interaction between the species involved in the electrodeposition and the LLC template, leading to a partial loss of horizontal orientation of the LLC during Pt nucleation before vertical rearrangement of the micelles to the electrode surface. The vertically aligned mesopores in the Pt and the possibility to produce freestanding films make these materials interesting in fields such as electrocatalysis, energy harvesting, and nanofluidics.

## Introduction

Lyotropic liquid crystal (LLC) templating is a versatile and facile way of producing mesoporous materials.^1–3^ The LLCs are self-assembled surfactants and can act as a soft template also for deposition.^4^ Deposition techniques include both chemical^1,5^ and electrochemical^6,7^ deposition. LLC templating has been applied on a variety of materials, *e.g.*, silica,^3,8,9^ metals,^6^ metal oxides like TiO_2_,^10^ semiconductors,^11^ and carbon.^12^ In recent years, the focus has shifted to LLC electrochemical deposition of mesoporous metals such as Pt, Pd or Ni, as they offer both electrocatalytic properties and high active surface area.^13,14^ The combination of large pore channel diameters (5–100 nm) compared to traditional porous materials or MOFs (<3 nm) and tunable pore morphology^4^ make mesoporous metals interesting in a wide range of applications, such as sensors,^15–17^ fuel cells, *e.g.*, as catalysts for the oxygen reduction^18–21^ and alcohol oxidation reaction^22^ or the hydrogen evolution reaction.^23^ For this purpose, a protocol for reproducible preparation of mesoporous Pt, Au, or Pd has been published.^24^

Mesoporous Pt films with hexagonal pore arrangement by templated electrodeposition were first reported by Attard *et al.*(6) Since then, many studies have been devoted to control of the pore morphology of the electrodeposited Pt film: the effect of the molar mass of the surfactant on diameter and center-to-center distance of the pores has been extensively studied by Wang *et al.*(25) and Asghar *et al.*(26) Takai *et al.*(27) produced various mesostructures, *i.e.*, two-dimensional (2D)-hexagonal, lamellar, and micellar cubic arrangements, by varying the composition ratio between block copolymers and aqueous Pt species. Asghar *et al.*(28) deposited 2D-hexagonal Pt films with pores having preferential orientation parallel to the substrate surface by applying shear stress to the LLC electrolyte before electrodeposition. Despite the preferential in-plane pore alignment evidenced by grazing incidence small-angle X-ray scattering (GISAXS), a large fraction of the inner pore surface was shown to be electroactive.

Generally, these studies suggest that the pores in the film inherit the structure of the LLC template. However, little is known about the growth of the mesoporous film, *i.e.*, the transfer of the nanostructure from the LLC template to the resulting film. Li *et al.*(4) suggest that LLCs undergo plastic deformation during the metal reduction reaction, proposing optimized reduction rates. Still, they fall short in describing the underlying phenomenon of plastic deformation of the LLC and its impact on the morphology of the electrodeposited film.

Up to now, common methods to study the film structure *ex situ*, *i.e.*, after electrodeposition and removal of the template/electrolyte, include SEM to study the overall film morphology as well as TEM and SAXS to study the structure of the mesopores.^1,27,28^ Generally, TEM measurements require specific preparation protocols (freestanding thin films or lamellae) for the sample to be able to resolve features such as mesoporous structures and are not statistically robust. SAXS on the other hand, probes a large representative surface area, which makes it suitable for characterizing long-range ordered structures, such as the mesopores of metal films electrodeposited from LLC templates.^27,29^ Due to its surface sensitivity, GISAXS has proven to be practical for studying surface morphology, *e.g.*, after nucleation and growth of Au particles,^30^ and preferential alignment of the pores in mesoporous films.^28,31^

Being applicable for *in situ* investigations, SAXS has been used to study Ag nanoparticle formation during multipulse electrodeposition.^32^ In our previous work, we investigated electrochemical aging by *in situ* GISAXS and observed changes in the morphology of fuel cell electrodes during simulated operation.^33–35^ A limited number of *in situ* SAXS studies during templated electrodeposition have been reported in literature. Richardson *et al.*(36) studied the templated electrodeposition of Pt films using phytantriol in inverse bicontinuous lipid cubic phases. The resulting morphology of the films had face-centered (*Fd*3*m*) symmetry with double the unit cell size of the primitive unit cell of the lipid template (*Pn*3*m*).

In the present work, the templated electrodeposition of Pt is studied for the first time by *in operando* GISAXS. An inherent reorientation of the LLC during electrodeposition is observed, which leads to Pt films with hexagonally arranged cylindrical mesopores aligned perpendicular to the electrode surface. This pore alignment has been shown to provide superior electrocatalytic properties due to the full accessibility of their large surface area.^31^

Subtle structural differences between the template and the mesoporous Pt film permit simultaneous monitoring and distinguishing structural changes of the template and of the mesoporous film. The combined approach of studying the changes of the surface morphology and the pore structure with *in operando* GISAXS allows correlating the nucleation and growth of the Pt film and structural changes of the LLC. Additionally, we demonstrate how to produce freestanding mesoporous Pt films, which makes them interesting in a variety of fields including energy harvesting^37^ or nanofluidic devices.^38^

## Experimental Section

### Chemicals for GISAXS Measurements

Hexachloroplatinic acid (HCPA) hydrate (99.9% trace metals basis, Sigma-Aldrich), Brij56 (decaethylene oxide monooctadecylether, C_18_EO_10_, Sigma-Aldrich), octaethylene glycol monohexadecyl ether (C_16_EO_8_, 98%, Fluka), KCl (potassium chloride, Sigma-Aldrich), and KCN (potassium cyanide, Sigma-Aldrich) were all used as received. For aqueous solutions, deionized (DI) water (18 MΩ, Milli-Q, Reptile Bioscience Ltd., Boston, MA) was used. All glassware used was washed thoroughly at least 3 times with DI water prior to use.

### Preparation of Electrolytes

For preparation of the electrolyte with hexagonal H_1_ LLC, Brij56 was mixed with 0.2 M aqueous (DI water) H_2_PtCl_6_ solution in 50:50 wt %. The LLC electrolyte for FIB and TEM samples consisted of C_16_EO_8_ and 2 M aqueous (DI water) H_2_PtCl_6_ solution in a 58:42 wt % ratio mixture. The surfactant mixtures are highly viscous and must be prepared with care to ensure a uniform composition. Therefore, the surfactants were heated to 50 °C prior to mixing to attain lower viscosity and then stirred with a glass rod for 30 min. Then, the sample vial was sealed airtight, and the mixture was left to equilibrate for 1 day. All of the resulting mixtures were checked by polarized light microscopy (Zeiss Stemi SV6, Carl Zeiss AG, Germany) for homogeneity and to ensure that it was in the hexagonal phase before use.^39^

In order to study the behavior of the LLC and the electrode surface upon application of the deposition potential but without film deposition, the LLC electrolyte was prepared without H_2_PtCl_6_, *i.e.*, 0.2 M aqueous (DI water) KCl solution and Brij56 in 50:50 wt %. KCl was added to attain ionic conductivity of the electrolyte.

### Electrochemical Deposition

**Figure 1:**
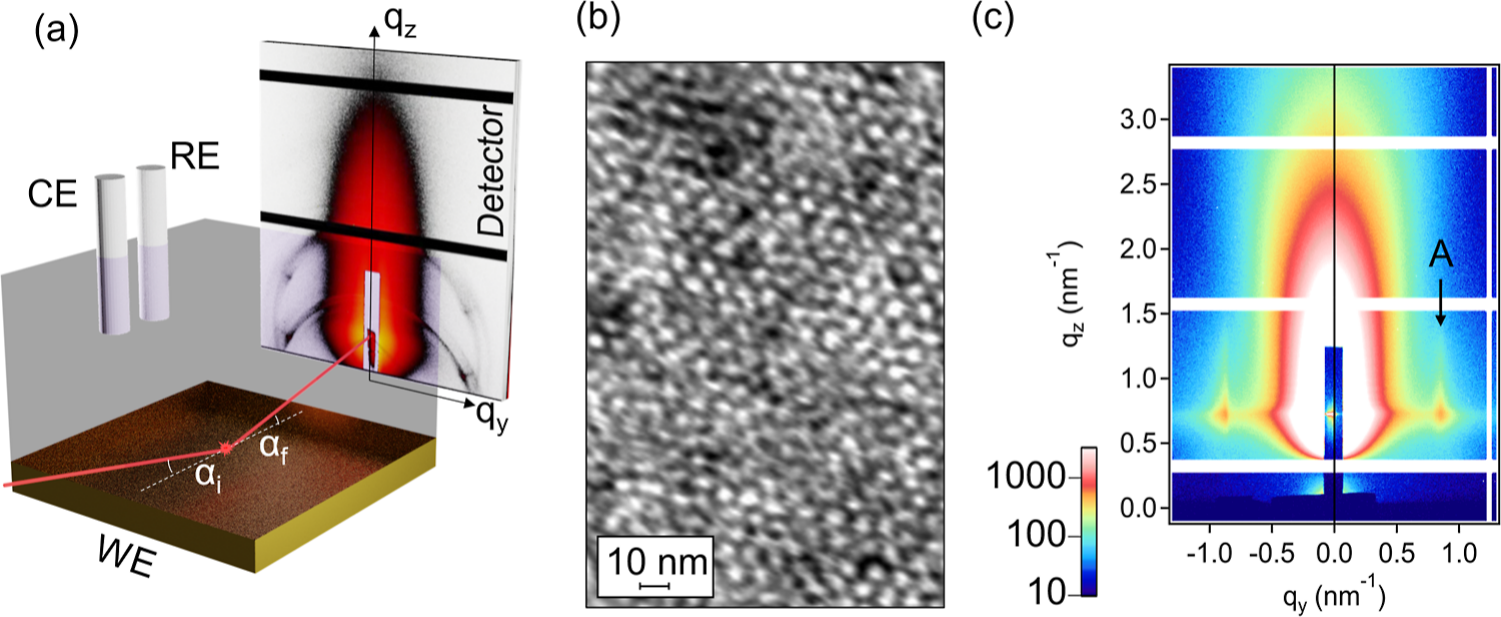
Operando GISAXS experiment during templated electrodeposition of H_1_-e Pt films: (a) Schematic experimental setup including three-electrode configuration with Au-coated silicon wafer as working electrode (WE), Pt wire as counter electrode (CE), and an Ag/AgCl-reference electrode (RE). The X-ray path across the electrolyte containing the LLC phase is indicated in red, impinging at the glancing angle α_i_. (b) Transmission electron micrograph of a freestanding H_1_-e Pt film electrodeposited on Au-coated mica, lifted off the substrate by dissolution of the Au layer in aqueous KCN. (c) GISAXS pattern of a mesoporous Pt film after deposition and removal of the electrolyte, intensity in arbitrary units. Vertical streaks (A) indicate the vertical preferential orientation of the pores in the Pt film.

Electrochemical measurements were performed with a potentiostat SP-240 from Biologic (Biologic Science Instruments GmbH, Göttingen, Germany). A custom-made electrochemical cell was used for *in situ* GISAXS during electrodeposition. The cell was made of Teflon with X-ray windows of Kapton. A schematic representation of the experiment is shown in Figure 1a, and an image of the cell is shown in the Figure S1. Due to its high viscosity, the electrolyte could be shaped using pistons to reduce the horizontal thickness of the electrolyte pillar to the thickness of the counter electrode, a Pt wire of 0.5 mm diameter. This enabled a short X-ray path through the electrolyte, reducing its absorption to attain an X-ray transmission of approximately 0.5 for an X-ray energy of 8 keV. The electrochemical deposition was performed in a three-electrode configuration, including an Ag/AgCl/KCl (3 M) reference electrode (Metrohm Herisau, Switzerland), a Pt counter electrode, and the deposition substrate as the working electrode. The deposition substrate was an Au layer of 50 nm thickness, sputtered on Si-wafer, with an 8 nm thick chromium adhesive layer. The deposition was performed at a constant potential of −0.069 V *vs* the Ag/AgCl reference electrode.

### FIB Sample Preparation and Imaging

Samples for FIB cutting were prepared by electrodepositing an approximately 3 μm thick H_1_-e Pt film from the template mixture on to gold electrodes (area 1 cm^2^) at a potential of −0.1 V *vs* a saturated calomel reference electrode (Schott, Germany) using a Pt gauze counter electrode. Gold electrodes were prepared by evaporation of a 10 nm thick chromium adhesion layer followed by 200 nm of gold on 1 mm thick glass slides. These evaporated gold electrodes were cleaned in an ultrasonic bath of 2-propanol for 10 min immediately before use. A DualBeam (SEM/FIB) Nova 200 NanoLab (FEI Company, Eindhoven, The Netherlands) was used to mill cross sections into electrodeposited H_1_-e Pt films.

### TEM Sample Preparation and Imaging

It turned out that the thickness of the H_1_-e Pt samples suitable for TEM analysis should not exceed 30 nm. Films were thus deposited for a period of 20 min onto Au-coated mica (20 × 20 mm^2^, Phasis Sàrl, Geneva, Switzerland) following the same procedure as described above for the FIB samples. The flame-annealed Au surfaces on mica are known to consist of large atomically flat Au(111) terraces. After the excess of the template had been rinsed off with water, the sample was placed into a saturated aqueous solution of KCN. After 1 day, the Au layer had been dissolved, and the thin H_1_-e Pt film had lifted off the substrate floating in the solution. From there, it could easily be collected onto TEM grids. The freestanding H_1_-e Pt films were studied with a TEM CM20 (Philips/FEI, 200 kV accelerating voltage, LaB6 cathode, Gatan imaging filter with 1k × 1k camera). Images are zero-loss filtered (with only elastic contribution from a 10 eV energy window). To estimate the pore center-to-center distance from the TEM images, 200 measurements were made using software ImageJ, and the mean and standard deviation were calculated.

### GISAXS Measurements

*In situ* GISAXS patterns were recorded at the Austrian SAXS beamline at the ELETTRA synchrotron in Trieste, Italy,^40^ with an incident X-ray beam with a wavelength of 1.54 Å (8 keV). The Au substrate acting as the working electrode for electrochemical deposition was positioned horizontally, as shown in Figure 1a. The direct and specular reflected X-ray beam, indicated as a red line, traverses the electrolyte close to the electrode surface at a grazing angle α_i_. The resulting scattering pattern is recorded by a 2D pixel detector (Pilatus3 1 M, Dectris Ltd.). The sample–detector distance was 118.1 cm, and the X-ray beam size was 0.1 × 2 mm (*V* × *H*), which allowed for a *Q*-range of 0.06 to 4.16 nm^–1^, determined with silver-behenate as a reference pattern. The cell was fixed at a glancing angle of 0.51° to the electrode surface (α_c,gold_ = 0.56°),^41^ which allowed us to measure the scattering pattern of the vertically aligned pore structure, partially superposed by the XRD rings from the LLC electrolyte. Operando measurements of the GISAXS patterns during electrodeposition were done with an exposure time of 5 s (7 s exposure period), scanning over 3 positions on the sample surface at an incremental distance of 2 mm, yielding a temporal resolution of 21 s for a single position. Before *ex situ* GISAXS measurements (after electrodeposition), the samples were washed thoroughly with deionized water to remove the electrolyte and LLC template residuals.

### GISAXS Analysis

For analysis of the scattering patterns, various data reduction protocols from 2D to 1D were applied to the GISAXS images: horizontal cuts at *q*_*z*_ = (0.78 ± 0.05) nm^–1^ (Figure S2) and (1.41 ± 0.08) nm^–1^ (Figure S3) were performed using software SAXSDOG.^42^ Azimuthal cuts (Figure S4) were done using software DPDAK.^43^ The software Fit2D was used for calibration.^44^ The intensity of the azimuthal cuts was normalized to the intensity of the incoming beam (ionization chamber). Data fitting of the 1D SAXS curves was performed with IGOR Pro (IGOR Pro 7.0.8.1, Wavemetrics).

Peaks in the azimuthal cut (Figure S4) are attributed to Bragg diffraction of the LLC phase aligned parallel to the sample surface. The horizontal cut at *q*_*z*_ = 0.778 nm^–1^ contains a peak at *q*_*y*_ ∼ 0.8–1.0 nm^–1^, attributed to (i) the 2D-hexagonal structure of the LLC in the electrolyte or (ii) the formation of structures aligned perpendicular to the sample surface. In both azimuthal and horizontal cuts, the peaks were fitted with a Lorentzian peak with linear background BG, yielding , with intensity *I*_Lor_, peak position *q*_0_, and full width at half-maximum (fwhm), and background (BG).

The horizontal cut at *q*_*z*_ = 1.41 nm^–1^ was performed to analyze the diffuse scattering of the electrode surface (Figure S3a). The high *q*_*z*_ was selected to avoid superposition with the pattern from the ordered pore structure and the vertical absorber stopping the total reflected beam, which allowed analyzing the low *q*_*y*_ region.

A first qualitative analysis was carried out by calculating a scattering invariant estimate *Q̃*_*y*_ of the horizontal cut.^45–47^*Q̃*_*y*_ was calculated over a limited regime in the in-plane direction, namely, *q*_*y*_ = 0.02 to 0.3 nm^–1^.

For a quantitative analysis, the focus is set on the relative changes in the structural parameters. As a consequence, we have used a simplified analytical model to interpret the horizontal cuts at *q*_*z*_ = 1.41 nm^–1^ rather than the full application of the distorted wave Born approximation (DWBA).^48^

The surface roughness of the electrode (Figure S3) was fitted with an analytical model, with *I*(*q*) = *I*_S_*P*(*q*_*y*_)*S*(*q*_*y*_) as a product of the scaling factor *I*_S_, the form factor *P*(*q*_*y*_), and the structure factor *S*(*q*_*y*_). As form factor *P*(*q*_*y*_), we chose a set of polydisperse spheres following the Schulz distribution *D*_n_(*R*),^49,50^ yielding *P*(*q*_*y*_) = |*f*(*R*_P_, σ_P_, *q*_*y*_)|^2^. The Schulz-sphere form factor allows the determination of the average particle radius, *R*_P_, and its standard deviation, σ_P_.

As structure factor, we chose the sticky-hard-sphere model,^51,52^*S*(*q*_*y*_) = *f*(*R*_SHS_, ϕ_SHS_, λ, ε, *q*_*y*_), which allows us to calculate minimum interparticle distance *d*_p,min_ = 2·*R*_SHS_ and to monitor the evolution of the apparent volume fraction, ϕ_SHS_, which is related to the probability of finding a nearest neighbor particle. The width of the square well, λ, was fixed to 0.1 in order to prevent linear dependence, and the “stickiness”, ε, remained constant at *ca.* −0.5 during fitting. The analytical model is described in more detail in our previous work,^33^ where this simplified description was developed and validated.

An additional Schulz-sphere form factor term *P*_2_(*q*_*y*_) was added to account for a shoulder evolving at *q*_*y*_ ∼ 2 nm^–1^, yielding the final formula *I*(*q*_*y*_) = *I*_S_*P*(*q*_*y*_)*S*(*q*_*y*_) + *I*_S2_*P*_2_(*q*_*y*_) + BG with background BG.

### Transmission SAXS Measurements

*Ex situ* transmission SAXS patterns of the mesoporous Pt film electrodeposited on Au/Si were recorded at the SAXSpoint 5.0 (Anton Paar GmbH, Graz, Austria) using a Mo source with an X-ray wavelength λ = 0.7107 Å (17.45 keV). The sample–detector distance was 160.63 cm, which allowed for a *Q*-range of 0.10 to 3.98 nm^–1^, determined with silver-behenate as a reference pattern. The samples were mounted with the electrodeposited side facing toward the detector; the pattern was recorded with an exposure time of 600 s.

## Results and Discussion

*Ex situ* transmission electron micrographs of mesoporous Pt (H_1_-e Pt) films electrochemically deposited from an LLC template show highly ordered cylindrical pores in 2D hexagonal arrangement (Figure 1b) with Pt walls, which is in accordance with literature.^6^

*Ex situ* GISAXS measurements (Figure 1c) show vertical streaks (highlighted and labeled “A”) reflecting the H_1_ ordering perpendicular to the sample surface, with a *d*-spacing of 7.4(4) nm derived from the (10) peak position. This corresponds to a lattice constant, *i.e.*, a center-to-center distance of the pores of 8.5(3) nm, which is in good agreement with the center-to-center distances of the pores in the TEM images (8.1(6) nm; Figure 1b), which were deposited under similar conditions. Regarding pore orientation, TEM shows the coexistence of domains of varying orientation in-plane and out-of-plane (Figure S5b in Supporting Information). However, *ex situ* GISAXS patterns, which show structural characteristics over a larger electrode area compared to TEM (2 mm × 0.5 mm in GISAXS compared to up to 30 μm^2^ in TEM), revealed no random orientation but predominantly vertically oriented pores. Additional SAXS measurements in transmission of the dry sample (Figure S6) confirm the center-to-center distance of the pores with a *d*-spacing of 7.42(3) nm, in good agreement with the vertical streaks measured in GISAXS [*d*-spacing 7.4(4) nm]. The equal *d*-spacings for SAXS and GISAXS measurements proves the preferential vertical orientation of the pores.

Before electrodeposition, a constant open-circuit potential of 0.532(1) V *vs* Ag/AgCl was measured for 10 s. In the hexachloroplatinate ion [PtCl_6_]^2–^, Pt has an oxidation state of +4. The electrochemical reduction process to elemental Pt occurs in two steps, from hexachloroplatinate [PtCl_6_]^2–^ to tetrachloroplatinate [PtCl_4_]^2–^ to elemental Pt^53,54^
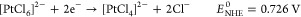
1
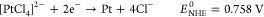
2

As the Pt species exists in 3 different oxidation states at the electrode surface, comproportionation/disproportionation may occur^55,56^
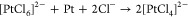
3

**Figure 2:**
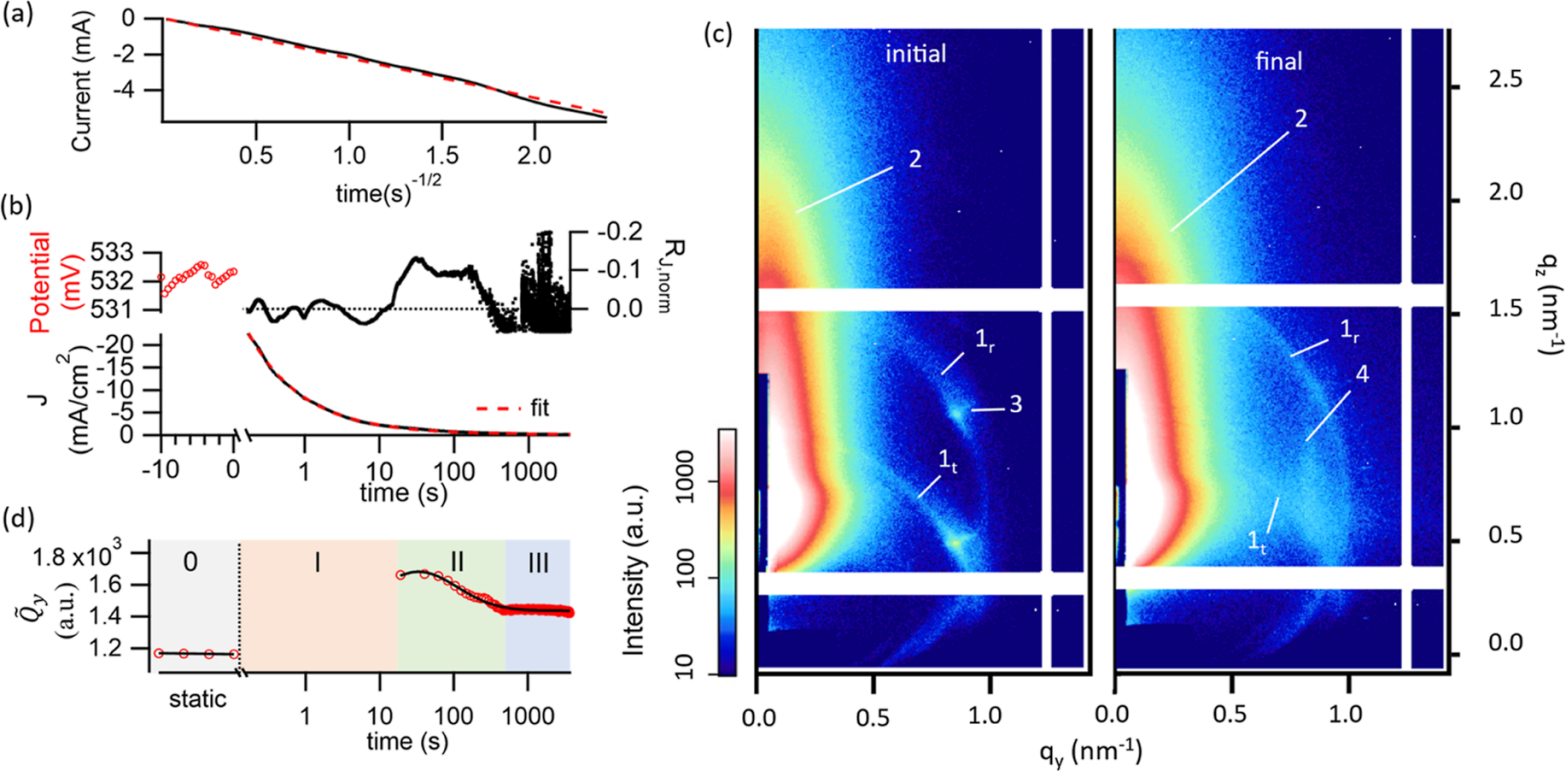
Results from chronoamperometry and GISAXS: (a) Cottrell plot of the chronoamperometric current. The linear trend, shown by a dashed regression line, indicates that the deposition is diffusion-controlled. (b) Open-circuit potential and chronoamperogram. The current density deviation *R*_J,norm_ is the fit residual of the power law fit of *J*, normalized by *J*, and is interpreted as a deviation from the diffusion-controlled behavior. (c) *In situ* GISAXS scattering patterns before (initial) and after electrodeposition (final). Several features of the scattering patterns are highlighted: (1) two powder diffraction rings of the LLC template (1_t_) from the direct beam and (1_r_) from the reflected indicate random orientation of the LLC. (2) Diffuse scattering of the electrode surface. (3) Diffraction peaks from LLCs with preferential orientation parallel to the sample surface. (4) Vertical streaks indicate structures with preferential alignment perpendicular to the sample surface. (d) Invariant estimate *Q̃*_*y*_ of the horizontal cut of diffuse scattering (2) shown in (c), plotted *vs* deposition time. Varying trends during the deposition process indicate several consecutive stages (0, I, II, and III).

To reveal the dynamics of interaction between the LLC template and the mesoporous film formation, electrochemical deposition in the current work was performed at a constant potential of −0.069 V *vs* Ag/AgCl. The Cottrell plot in Figure 2a indicates a largely diffusion-controlled electrode reaction, as shown by the linear trend.^57^ Linear regression yielded a fit quality of reduced χ^2^ ∼ 483, determined by Pearson’s χ-squared test.

For a comparison of the current density *J* with morphological changes during templated electrodeposition, *J vs* time was fitted with a power law (Figure 2b) and the Cottrell equation (Figure S7). Fitting with the Cottrell equation yielded a diffusion coefficient of the hexachloroplatinate of 4(1) × 10^–8^ cm^2^/s. Similar values (4–5 × 10^–8^ cm^2^/s) were derived by Reiter *et al.*(58) for aqueous ferrocene solutions in a 50 wt % mixture with Brij56. The diffusion coefficients being lower by a factor of 6–14 compared with those in liquid solvents were attributed to restricted mobility and channel size effects in the LLC. The goodness of the Cottrell equation fit, determined by Pearson’s χ-squared test, gave a reduced χ^2^ ∼ 548, whereas the power law resulted in a better fit (reduced χ^2^ ∼ 108). The power law fit of *J* (Figure S8) yielded an exponent of time of −0.52, very close to −0.5 expected for diffusion-limited behavior of the reaction as described in the Cottrell equations.^57^ The fit residual *R*_J_ is interpreted as a deviation from the diffusion-controlled behavior. To attain a more physical meaning, *R*_J_ was normalized by current density *J*. For comparison, the current density *J* and the residuals *R*_J_ and *R*_J,norm_ are shown in Figure S9, *R*_J,norm_ is shown on top of current density *J* in Figure 2b. The initial peak is attributed to the charging current of the electrochemical double layer, which decays within 200 ms and is not accounted for by the power law. The subsequent negative deviations resulting in several peaks can be attributed to nucleation and therefore can be assigned to structural changes detected by GISAXS. A limitation of this assignment is that the chronoamperometric curve represents the integral signal of the entire electrode surface (*ca.* 24.6 mm^2^), whereas GISAXS probes only a strip of 0.5 × 2 mm^2^.

*In situ* GISAXS patterns before and after electrodeposition (Figure 2c) show features from both the electrolyte and the electrode surface: generally, (1) the XRD ring with a *d*-spacing of 6.5(3) nm is the signature of the randomly oriented LLC in the electrolyte, which consists of 2 contributions from the transmission (1_t_) and reflection channels (1_r_) of the DWBA theory,^48^ (2) the diffuse scattering from the electrode surface, which resembles the pattern of sputtered Au.^59^ Initial and final pattern have features indicating different structures: (3) spots in the initial pattern indicating preferential orientation of the LLC parallel to the electrode surface,^60^ and (4) vertical streaks indicating preferential orientation perpendicular to the electrode surface.^61^

The *d*-spacing of the vertical streaks (4) is higher than that of the spots of LLC (1), at 7.4(4) *vs* 6.5(3) nm (Figure 1c), indicating a center-to-center distance of the pores in the deposited Pt film higher than that of the micelles in the LLC [8.5(5) *vs* 7.5(3) nm]. The streaks (4) are even more prominent in the pattern of the dry sample, *i.e.*, after deposition and subsequent removal of the template (direct comparison to *in situ* measurements in Figure S10).

From the point of view of GISAXS, it is important to stress that the samples *in situ* can be considered as composite of two semi-infinite layers, namely, the Au electrode and the electrolyte containing the LLC, where the electrolyte is being traversed by the X-ray beam (Figure 1a). As the electrodeposition proceeds, we monitor (i) the formation of the mesoporous Pt film and (ii) the changes in the LLC close to the electrode surface. While process (i) is surface sensitive, the LCC is measured to a maximum distance of *ca.* 100 μm into the electrolyte (essentially the vertical X-ray beam size). This distance is much larger than the distance of the reduction process being responsible for changes of the LLC (ii), which has been estimated with 20 μm from the diffusion length after 30 s with a diffusion coefficient of 4(1) × 10^–8^ cm^2^/s. Therefore, we suggest that the signals from the LLC primarily come from regions that are not affected by the electrodeposition process, *i.e.*, random oriented LLC. Only a minor contribution come from the preferential parallel orientation of the LLC being close to the electrode surface.

Asghar *et al.*(28) described a similar system with the surfactant C_16_EO_10_ and an aqueous hexachloroplatinic acid solution to produce mesoporous Pt films with cylindrical pores in hexagonal arrangement oriented parallel to the sample surface. They attained preferential orientation of the pores on a ridged archival gold DVD-R disc as a working electrode by applying shear stress to the LLC electrolyte prior to electrodeposition. They suggested that the LLC phase was aligned with the electrode surface by shearing, and during the subsequent electrodeposition, the pores in the Pt film inherited the preferential orientation from the LLC, as evidenced by *ex situ* GISAXS. In our current work, *in situ* GISAXS patterns before and after electrochemical deposition show loss of preferential orientation of the LLC. Furthermore, we see alignment of the final pore structure vertical to the electrode surface. Both the loss of preferential orientation of the LLC and the vertical alignment of the pore structure suggest a mutual interaction between the deposition process and the LLC template, which was not observed by Asghar *et al.*(28) The reason could be that their Au working electrode was ridged, *i.e.*, it had grooves with a repeating distance of *ca.* 0.5 μm and a depth of *ca.* 0.1 μm. In the case of a redirecting effect by the electrode, these grooves would suppress streaks coming from vertically aligned structures in the GISAXS pattern. Indeed, they did not observe any preferential orientation when they deposited the films without aligning the LLC prior to electrodeposition. For our measurements, in contrast, the working electrode was an Au layer of 50 nm thickness, sputtered on a Si-wafer, with an 8 nm thick chromium adhesive layer.

Asghar *et al.*(26) reported no significant differences in the LLC template and the pore distance of the electrodeposited film using similar systems, *i.e.*, Brij-56 and aqueous HCPA solutions. In our case, the *d*-spacing of the vertical streaks is significantly higher at 7.4(4) *vs* 6.5(3) nm, which is directly visible in the *in situ* pattern after electrodeposition (Figure 2c).

In order to study subtle structural changes of the electrode and the LLC during electrodeposition, we performed *in operando* GISAXS measurements scanning over 3 positions of the electrode in raster fashion with an exposure time of 5 s and an exposure period of 7 s, yielding a temporal resolution of 21 s. A time-lapse movie of the detector images during electrodeposition at one position is presented as Supporting_Video_1.

**Figure 3:**
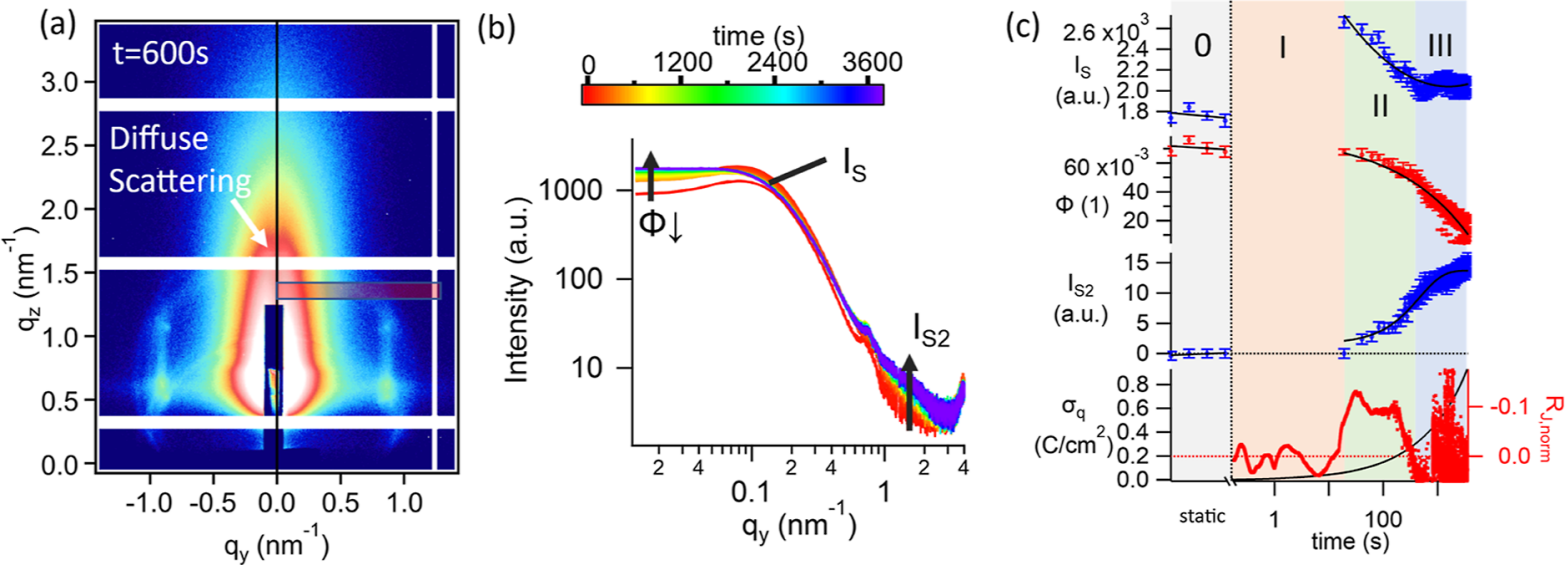
Horizontal cut of diffuse scattering: the cut/integrated region is indicated by a red rectangle in (a), the resulting 1D patterns are shown in (b). *In operando* patterns during electrodeposition are shown with a rainbow color code, and trends in the scattering patterns are labeled, referring to fit parameters shown in (c). The temporal evolution of the surface morphology, as indicated by fit parameters from the horizontal cut of the diffuse scattering pattern, is shown in (c): the scaling factor *I*_S_ of the interacting spherical particles and its particle volume fraction ϕ derived from the sticky-hard-sphere structure factor are nonzero at stage 0, *i.e.*, *I*_S_ and ϕ initially represent the roughness of the Au electrode surface. The increase in *I*_S_ at stage I indicates that the structure of the deposited Pt replicates the Au structure. The time resolution of the GISAXS measurements (21 s) allowed us to monitor only initial and final states during stage I of electrodeposition. In stage II, *I*_S_ and ϕ decrease and a new structure emerges, represented by the second Schulz-sphere term with intensity *I*_S2_. The transferred charge density σ_q_ and the current density deviation *R*_J,norm_, normalized by the current density *J*, calculated from the diffusion model, are shown at the bottom for comparison.

For a first qualitative assessment of the structural evolution at the electrode surface during electrodeposition, the diffuse scattering (2) was analyzed by performing a horizontal cut (shown in Figure 3a) and calculating an invariant estimate *Q̃*_*y*_, shown in Figure 2d. The invariant estimate *Q̃*_*y*_ indicates several stages with varying trends over time, labeled 0 before deposition, and I, II, III during electrodeposition.

The diffuse scattering peak is the signature of the electrode surface. Using cuts in-plane or out-of-plane, vertical and horizontal structural information (*i.e.*, the morphology along directions perpendicular and parallel to the electrode) are decoupled. The horizontal cut (Figure 3a) enabled us to study the surface structure by conventional model fitting, with Schulz-sphere form factor and sticky hard sphere structure factor (Figure 3b). Previous studies have shown that this is sufficient to analyze trends of structural changes on the sample surface.^33^

The intensity *I*_S_ (in Figure 3c) is nonzero before starting the deposition, *i.e.*, initially, the signal derives from the surface morphology of the Au substrate. The Schulz-sphere form factor, indicating spheres with Schulz-distributed diameter, yields a mean diameter of 1.5 nm (sigma 2.5 nm) for the Au substrate, resulting in a median particle diameter of 12.6 nm, which is in line with previous studies on gold sputtered films by GISAXS.^59^ The sigma value is fixed for all data in the set to prevent the linear dependence of parameters.

In stage I of deposition, intensity *I*_S_ increases by 40–50%, with no change in structural parameters; *i.e.*, mean diameter and sigma remain constant. The time resolution of the GISAXS measurements (21 s) allowed us to monitor only initial and final state during stage I of electrodeposition. The strong increase in intensity indicates a nucleation burst of Pt on Au during stage I. The morphology of the Pt film is commensurate with the Au substrate; *i.e.*, the deposited Pt film has morphological features reflecting the Au substrate, as illustrated at the bottom of Figure 5 (stage I). Moehl *et al.*(30) reported that the correlation length varied during deposition of Au on a TiN substrate. On the other hand, studies by Yamauchi *et al.*(62) indicate that the atomic arrangements of the deposited Pt and PtAu reflect those of the Au substrate. They attributed this to both substrate and deposit having a fcc structure and a similar lattice constant, which promotes 2D-heterogeneous nucleation. Also, studies of electrodeposition of Pt on Au by Lin-Cai and Pletcher^63^ indicated an initial nucleation stage governed by the formation of one or a few monolayers of Pt, followed by three-dimensional growth.

The sticky hard sphere structure factor indicates an average interparticle distance of 46 nm. The particle volume fraction Φ, indicating the probability of two neighboring (sticky) particles, is nonzero initially and does not change at the start of deposition. During stages II and III, Φ decreases continuously. This indicates a decrease of the interparticle ordering, which is attributed to deposition of Pt with different structures in stages II and III.

A second form factor term for polydisperse spheres (sizes following a Schulz distribution) with intensity *I*_S2_ was fitted to account for a shoulder emerging at scattering vector *q*_*y*_ ∼ 1.8 nm^–1^. The evolution of the intensity *I*_S2_*vs* time is shown in Figure 3. The signal emerges in stage II, continuously increasing in stages II and III. The signal is attributed to evolution of a new morphology of the deposited Pt, consisting of spherical particles with Schulz-distributed diameter, mean diameter 0.8 nm (sigma 0.4 nm), resulting in a median particle diameter of 2.6 nm. Both the mean diameter and the sigma value are fixed for all fitted data. This surface morphology could be either attributed to the roughness of the inner walls (the wall thickness at the point of nearest contact between neighboring pores was estimated *ca.* 2.5 nm *via* TEM)^53^ or to the surface morphology of the Pt film.

For the purpose of comparison with the X-ray measurements, the normalized current density deviation *R*_J,norm_ is shown on the bottom of Figure 3. *R*_J,norm_ displays several peaks in stage I, which were not registered by GISAXS, having a temporal resolution of only 21 s.

**Figure 4:**
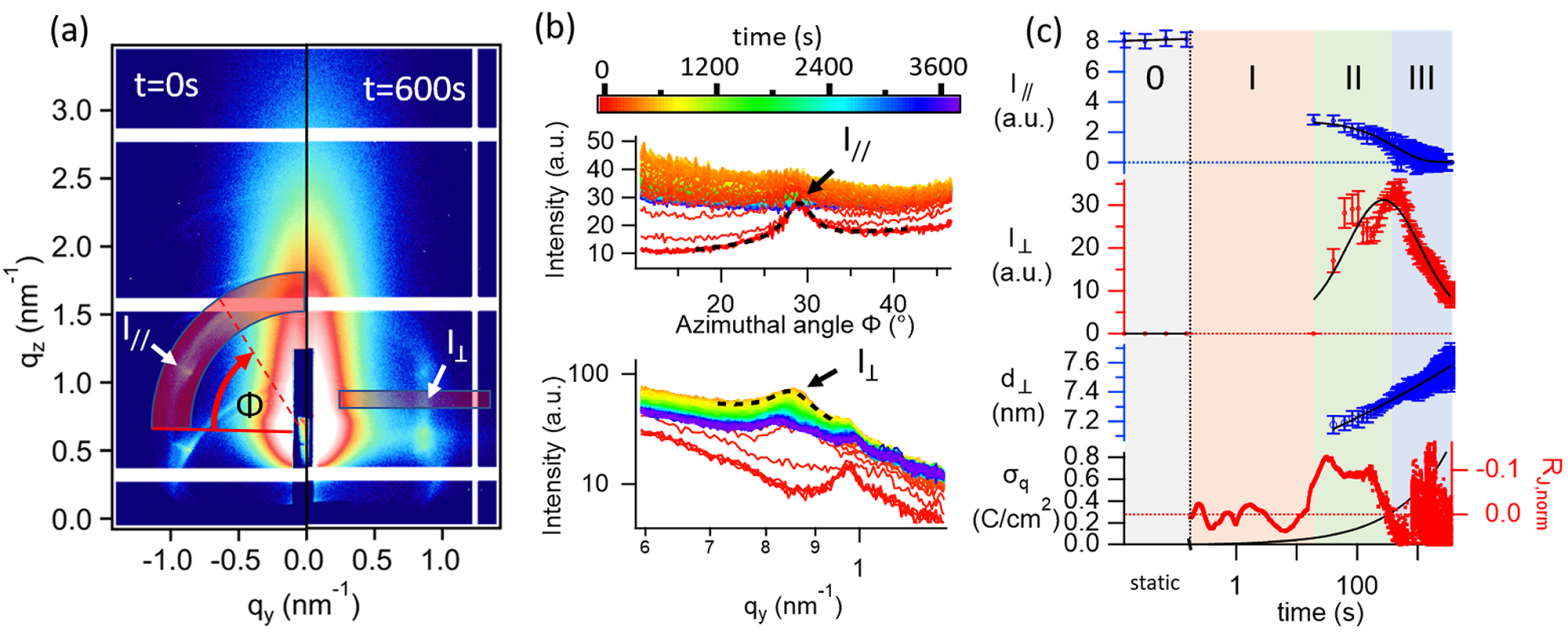
Azimuthal cut of the diffraction ring (reflection channel) and horizontal cut of the streak, performed on *in operando* scattering patterns: the cut/integration regions are highlighted in red (a), and resulting 1D patterns are shown in (b), labeling the peaks of the ordered structures with orientation parallel (∥) and perpendicular (⊥) to the electrode. The temporal evolution of ordered structures is shown in (c): intensity *I*_∥_ of the signal indicating a preferential orientation of the LLC parallel to the electrode surface, intensity *I*_⊥_ and *d*-spacing *d*_⊥_ of the mesopores aligned perpendicular to the electrode surface together with the consumed charge density σ_q_, and the fit residual *R*_J,norm_ normalized by the current density *J* from the power law fit of the current density *J*.

To study the temporal evolution of LLC with a preferential orientation parallel to the electrode surface, an azimuthal cut of the diffraction ring (reflection channel) was made. The cut was made integrating the 2D data radially between 0.766 nm^–1^ inner radius to 1.132 nm^–1^ outer radius, as shown in Figure 4a, on the left (*t* = 0 s). The resulting 1D pattern *vs* azimuthal angle Φ, shown in Figure 4b, having peaks at 60, 90, and 150°, attributed to the 2D hexagonal structure with preferential orientation parallel to the electrode, were fitted with a Lorentzian. The resulting temporal evolution of the peak intensity *I*_∥_ of the LLC with preferential orientation parallel to the electrode surface (labeled 3 in Figure 2c) is shown in Figure 4c. Static measurements show constant intensity *I*_∥_ before the deposition start (stage 0). During stage I, intensity *I*_∥_ decreases by about 70% [8.1(1) *vs* 2.8(2), in a.u.]. In stages II and III, it decreases continuously to less than 1% of the initial intensity. The decrease of intensity *I*_∥_ indicates a (partial) loss in preferential orientation of the LLC parallel to the electrode.

The driving force of the loss of preferential orientation is either (i) electric double layer charging or (ii) a change in the interaction between the LLC and the Pt species during deposition. Electric double layer charging (i) can be excluded based on chronoamperometry with the same potential and the LLC electrolyte containing no Pt salt. The measurements showed no changes of intensity *I*_∥_ (Figure S11), indicating that the loss of long-range order is driven by an interaction between the LLC and the Pt species during deposition.

Analogous to *I*_∥_, to analyze the signal from the mesopores in the Pt film oriented perpendicular to the electrode (labeled 4 in Figure 2c), a suitable cut region (highlighted as a red rectangle on the right side of Figure 4a) was selected to make a cut of the 2D scattering pattern. The peak in the resulting 1D pattern (Figure 4b, indicated with *I*_⊥_) was fitted by a Lorentzian, and its intensity *I*_⊥_ is shown in Figure 4c. The peak (*I*_⊥_) emerges at the beginning of stage II (after about 14 s of electrodeposition), *i.e.*, after the partial loss of preferential orientation of the LLC parallel to the electrode in stage I (feature labeled 3 in Figure 2c, intensity *I*_∥_ in Figure 4c). *I*_⊥_ then reaches a maximum at the end of stage II (about 420 s). At this stage, the Pt film thickness is estimated to be approximately 69 nm (Figure S12).

In stage III, the intensity *I*_⊥_ decreases continuously. The *d*-spacing *d*_⊥_ increases continuously during stages II and III [from 7.18(5) to 7.56(4) nm], indicating increasing center-to-center distance between the pores, *i.e.*, a swelling of the mesostructure, by *ca.* 5% between stages II and III of deposition [8.29(6) to 8.73(5) nm], which is attributed to growth of Pt walls between the micelles.

The duration of the stages was about 14 s for stage I, 420 s for stage II, and about 3100 s for stage III. The duration of stages II and III varied for the three different measuring positions, as shown in Figure S13. Stage III was not reached at all positions. This is attributed to locally varying deposition rates, *i.e.*, nonuniform current distribution.

The decrease in intensity *I*_⊥_ deriving from the vertical pore channels in stage III is attributed to a Debye–Waller-like behavior^64,65^ of deposition, characterized by local displacements of the LLC from their lattice position during electrodeposition. Due to the nonuniform deposition rates, local fluctuations of the deposition lead to local variations of the LLC ordering, while the overall long-range ordering in the LLC domain is maintained. This is in good agreement with previous studies by Elliot *et al.*,^53^ describing that the pore channels were not straight, but a degree of meandering could be observed using TEM images. Contributions from the form factor^66–68^ of the LLC on the decrease in intensity *I*_⊥_ were excluded by SAXS measurements of the LLC (as shown in Figure S14). Small variations in the *d*-spacing of the LLC can significantly change the intensity of the XRD reflections, if their position is close to a local minimum of the form factor (*e.g.*, at *q* ∼ 1.2 nm^–1^ in Figure S14c). However, from the form factor of the pore calculated for the present LLC, the intensity *I*_⊥_ is expected to increase with a higher *d*-spacing *d*_⊥_ of the mesopores.

**Figure 5:**
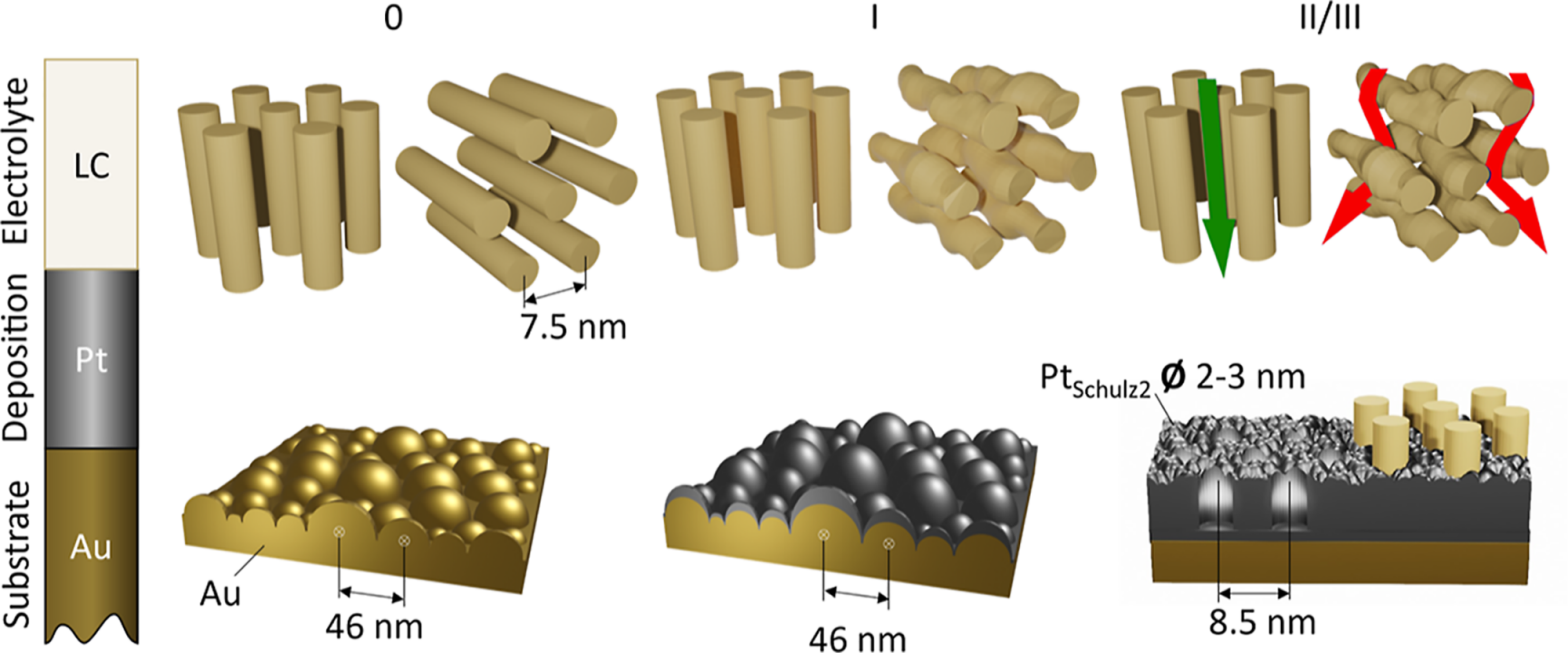
Schematic representation of structural changes of the electrode surface (bottom) and the LLC close to the electrode (top) during the deposition process: before Pt deposition (0), GISAXS indicates a polydomain LLC with random orientation and preferential orientation parallel to the electrode surface. In stage (I), governed by nucleation, the preferential orientation decreases and a Pt layer forms on the Au electrode, with a structure replicating the electrode. In (II), the deposited Pt film attains a structure with smaller roughness (Schulz sphere diameter 2–3 nm), and mesopores emerge with a center-to-center distance of 8.5(5) nm and a preferential orientation perpendicular to the electrode surface. The green and red arrows at the LLC in phases II/III illustrate the direction of mass transport in the electrolyte toward the electrode.

Structural changes of the LLC (Figure 5 top) and the electrode surface (Figure 5 bottom) are combined schematically in Figure 5 for stages 0 (before deposition) and I, II, and III (final stage) of deposition.

In stage I of electrodeposition, the loss of preferential orientation of the LLC and the confirmation by experiments without Pt salt suggest that the driving force is a change in the interaction between the LLC and the Pt species during deposition. At this stage, attributed to Pt nucleation, the deposition rates are the highest, and there is an abrupt local depletion of chloroplatinate species close to the electrode.

In stage II, the growth of the H_1_-e Pt film with vertically oriented pores indicates (i) preferential growth of Pt in domains with LLC oriented vertically or (ii) reorientation of the LLC. Preferential growth (i) can be due to higher mass transport/enhanced diffusion of hexachloroplatinate along LLC with vertical orientation because it is parallel to the concentration gradient. For domains with horizontally aligned pores, the interaction between the chloroplatinate species and the LLC is expected to reduce the rate of mass transport from the bulk electrolyte to the electrode surface, as indicated in Figure 5, stage II/III top. This is also reflected in the electrochemical data as there is a broad peak of the normalized current density deviation *R*_J,norm_ (Figure 4) in stage II, indicating increased Pt deposition rates. As the vertically aligned mesoporous structures reach a maximum (intensity *I*_⊥_ in Figure 4), *R*_J,norm_ decreases, indicating the depletion of the hexachloroplatinate in the vertical LLC domains. On the other hand, in case of (i), the film growth is expected to be inhomogeneous as the deposition rate varies between domains with different orientations of the LLC. SEM images (Figure S15) show homogeneous coverage of the film over an area with several domains of different orientations. Also, as indicated by intensity *I*_∥_ in Figure 4, there is a loss in preferential orientation of LLCs parallel to the electrode in stage I of deposition. Therefore, we suggest that the orientation of the LLC changes in several domains during deposition, from parallel with respect to the electrode surface to perpendicular.

There are studies about the change of orientation of liquid crystals in an electric field, *e.g.*, by adding an electroactive molecule^69^ or by functionalized liquid crystals.^70^ In the present study, the LLC itself is not electroactive, but the dissolved Pt species interact with the LLC during electrodeposition.

The interaction can be due to changes in the local chemistry of the aqueous domain, *e.g.*, by local accumulation of Cl^–^ (see eqs 1 and 2) or an inverse piezoelectric effect^71^ due to depletion of the hexachloroplatinate, leading to changes in the volume of the aqueous domain and consequently to local changes of *d*-spacing of the LLC. Takai *et al.*(27) reported that the relative size of the hydrophilic headgroup increases with the content of Pt precursors, which induces a stronger curvature of the LLC mesophases and, in turn, triggers mesoscopic phase change from lamellar to hexagonal or to cage-type. In static measurements, we could not see any changes of phase or *d*-spacing in LLC mixtures with and without. However, the pore *d*-spacing was significantly higher in the deposited Pt, compared to the *d*-spacing of the LLC, 7.4(4) *vs* 6.5(3) nm. The increase in *d*-spacing of the LLC is attributed to growth of Pt between the cylindrical micelles, leading to swelling of the mesophase. This indicates that the reorientation in our case is a dynamic effect triggered by an abrupt local composition change close to the electrode.

As the reorientation is directed perpendicularly to the electrode, the driving force should be directed the same. A possibility is the interaction of the Pt species during mass transport toward the electrode, deforming the LLC, *e.g.*, by coordination with the head groups of the amphiphiles^72^ in the LLC and dragging the amphiphiles with it. The abrupt local depletion of Pt species in stage I results in strong concentration gradients toward the electrolyte bulk, leading to diffusional mass transport of the Pt species toward the electrode. The diffusional rate of chloroplatinates is strongest at the initial stage of deposition and decreases as the concentration gradient decreases over time, in agreement with the decrease of intensity *I*_∥_, which is strongest at stage I of deposition.

Another mass transport mechanism apart from diffusion could be the migration of chloroplatinate ions in the electric field. However, migration should be a subordinate effect because (i) [PtCl_6_]^2–^ is negatively charged, yielding a negative force of the electric field on the ion. Also, (ii) in the case of migration of the chloroplatinate ion, the decrease in intensity *I*_∥_ should be continuous over time. In addition, (iii) there should be an effect visible in the control measurements without chloroplatinic acid, as there should be considerable migration of K^+^ and Cl^–^ ions during electric polarization.

To conclude, we suggest that during electrochemical deposition of H_1_-e Pt, the local depletion of chloroplatinate ions close to the electrode surface causes deformation of the LLC. Consequently, the LLC close to the electrode partly loses its structure. Due to the following mass transport of the chloroplatinate ions toward the electrode, there is a directing effect on the LLC causing a rearrangement of the LLC with preferential orientation perpendicular to the electrode surface. The resulting Pt film attains pores with a preferential orientation perpendicular to the electrode.

In stage III, the mesoporous film grows continuously at slower rates, as indicated by the chronoamperometric data. Debye–Waller-like behavior of the mesopores and variations of the duration of stages II and III measured at different positions indicate increased local variations of deposition/growth rates at later stages of deposition. However, the continuous increase of intensity *I*_S2_, attributed to the Pt surface morphology, indicates continuous growth of the mesoporous Pt film.

## Summary and Conclusions

The electrochemical deposition of mesoporous Pt from a hexagonal H_1_ LLC template was investigated by GISAXS. *In situ* GISAXS showed different preferential orientations of the LLC template and the mesopores of the electrodeposited Pt film. Before electrodeposition, the LLC template had a preferential orientation parallel to the electrode surface. In the electrodeposited mesoporous Pt film, the hexagonally arranged pores had a preferential orientation perpendicular to the electrode surface and an increased center-to-center distance compared to the LLC template [8.5(5) *vs* 7.5(3) nm].

In order to measure the structural evolution of mesoporous Pt films by *in operando* GISAXS during electrodeposition, a miniaturized electrochemical cell with a beam path length of 0.5 mm through the LLC electrolyte was developed, attaining X-ray transmission of approximately 0.5 for an X-ray energy of 8 keV.

*In operando* GISAXS measurements with a temporal resolution of 7 s show that the preferential orientation of the LLC film parallel to the electrode surface decreased at the beginning of electrodeposition, reaching a plateau after 14 s. The simultaneous nucleation burst of Pt indicates a mutual interaction between the electrodeposition process and the LLC template. We suggest that the local depletion of the chloroplatinate ions and accumulation of Cl^–^ in the diffusion layer during nucleation of the Pt film cause partial loss of long-range ordering of the LLC close to the electrode.

The nucleated Pt had a morphology commensurate with the Au substrate, *i.e.*, the deposited Pt film had morphological features reflecting the Au substrate (median particle size of 12.6 nm, determined by GISAXS), indicating 2D nucleation of Pt on Au.^63^ During the subsequent growth of the electrodeposited Pt film, the morphology changed toward smaller particles (median particle size of 2.6 nm) and hexagonally arranged mesopores emerged. They reached a maximum of long-range ordering after *ca.* 420 s of deposition, corresponding to a film thickness of approximately 69 nm, indicating rearrangement of the templating LLC close to the electrode with a vertical preferential orientation. At later stages of deposition, *in operando* GISAXS indicated increased local variations in growth rates.

These findings yield new insights into the LLC templated electrodeposition process, showing an inherent reorientation of the LLC during electrodeposition. This results in Pt films with hexagonally arranged cylindrical mesopores aligned perpendicular to the film surface, which are expected to provide superior electrocatalytic properties due to their low diffusion resistance, giving full access to their large inner surface area.^31^ Furthermore, the ability to lift these films off the substrate to produce freestanding films makes them interesting in a variety of fields, *e.g.*, energy harvesting^37^ or nanofluidic devices.^38^

## Supporting Information Available

Additional details regarding experimental and data analysis of the GISAXS patterns; photograph of the in situ electrochemical cell; transmission electron and scanning electron micrographs of the Pt film; additional experimental data of SAXS, GISAXS, and the chronoamperometric measurements (PDF)
Time-lapse data of the detector images during electrodeposition at one position (MP4)

## Abbreviations

LLC: lyotropic liquid crystal
GISAXS: grazing incidence small-angle X-ray scattering
HCPA: hexachloroplatinic acid
