# Supplementary material for: Monitoring of Pore Orientation by in Operando Grazing Incidence Small-Angle X-ray Scattering during Templated Electrodeposition of Mesoporous Pt Films
Source: ACS Appl Mater Interfaces. 2023 Sep 28;15(40):47604–14. doi: 10.1021/acsami.3c03316 (PMC10571001; doi:10.1021/acsami.3c03316)
Supplement: Supplementary file 1 — am3c03316_si_001.pdf [file am3c03316_si_001.pdf]

Supporting Information

Monitoring of Pore Orientation by In Operando Grazing Incidence Small Angle X-ray Scattering during Templated Electrodeposition of Mesoporous Pt films

Philipp Aldo Wieser[1], David Moser[2], Bernhard Gollas[3]‡, Heinz Amenitsch[1]*‡*

1. Institute of Inorganic Chemistry, Graz University of Technology, Graz, Austria

2. Institute of Electron Microscopy and Nanoanalysis, Graz University of Technology, Graz, Austria

3. Institute for Chemistry and Technology of Materials, Graz University of Technology, Graz, Austria

‡These authors contributed equally.

*Correspondence e-mail: bernhard.gollas@tugraz.at and amenitsch@tugraz.at

Contents

Electrochemical cell ... 2
Horizontal cuts of the GISAXS patterns ... 2
Azimuthal cut of GISAXS scattering pattern ... 3
Transmission electron micrographs of free-standing Pt films ... 4
SAXS measurement of Pt film in transmission ... 5
Cottrell fit of chronoamperometric data ... 5
Power law fit of chronoamperometric data ... 6
Comparison of Current density deviation to normalized current density deviation ... 7
In operando GISAXS measurements without Pt salt ... 8
Estimation of film thickness at maximum pore ordering (at ca. 420 s) ... 9
Fitting parameters at different positions ... 11
Contributions of the form factor of the LLC on the decrease in intensity $I_{\perp}$ ... 11
Scanning electron micrographs of Pt film ... 14
References ... 14

Electrochemical cell

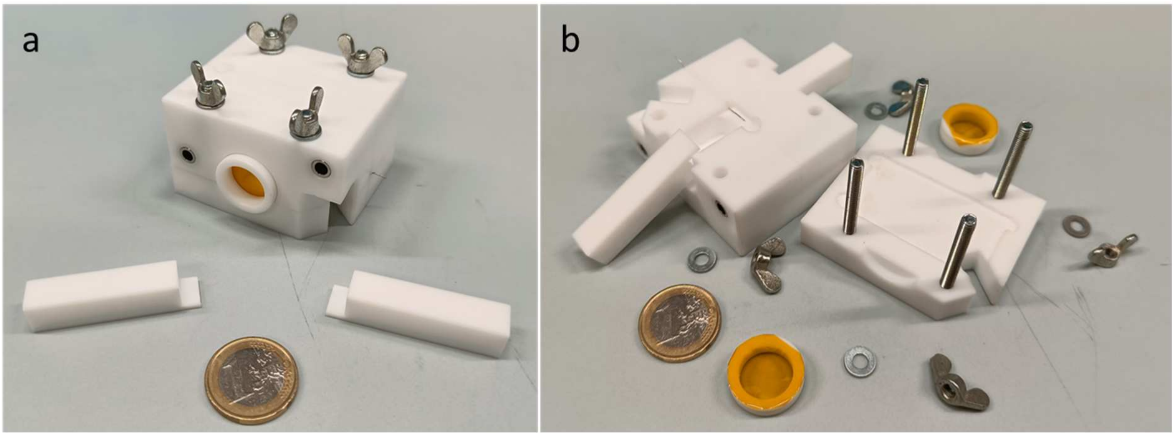

Figure S1: Electrochemical cell, (a) assembled with 2 pistons to shape the electrolyte on the electrode and (b) disassembled (without working and reference electrodes).

Horizontal cuts of the GISAXS patterns

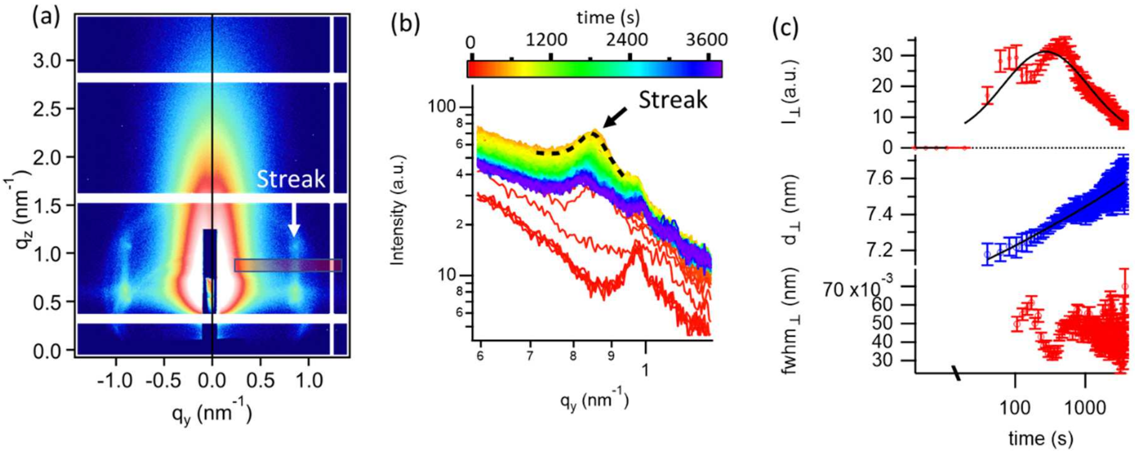

Figure S2: For analysis of the vertical streaks indicated with an arrow in (a), a horizontal cut was performed at $q_z = (0.78 \pm 0.05)$ nm^{-1}. The cut region for vertical streaks is shown as a rectangle in (a). Integrated Intensity vs q_y is shown in (b). The signal from the vertical streaks was fitted by with a Lorentzian with linear background, its temporal evolution is shown in (c).

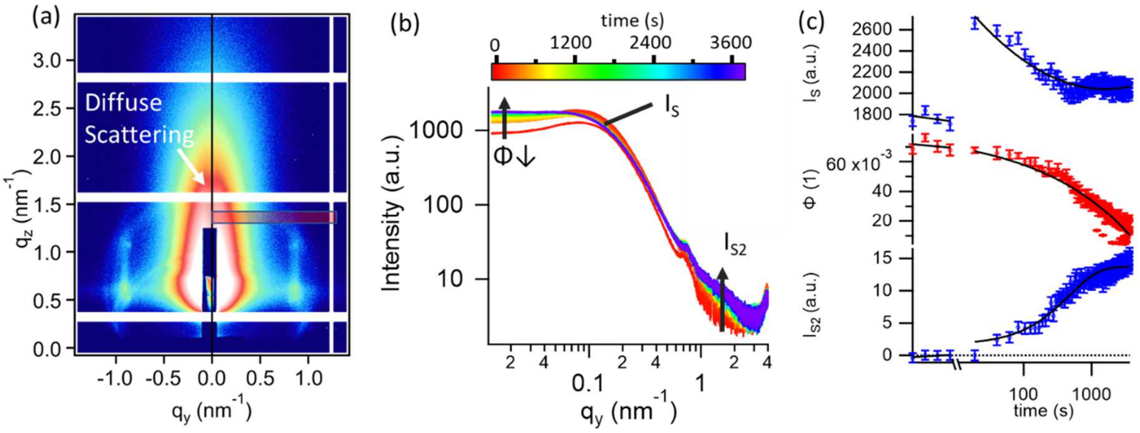

Figure S3: In order to analyse the diffuse scattering from the substrate surface, a horizontal cut was performed at q_z = (1.41±0.08) nm^{-1}, indicated by a rectangle in (a). Integrated intensity vs q_y is shown in (b). The signal from the vertical streaks was fitted with Schulz Sphere Form Factor and Sticky Hard Sphere Structure Factor (further explained in the experimental in the main text), its temporal evolution is shown in (c).

Azimuthal cut of GISAXS scattering pattern

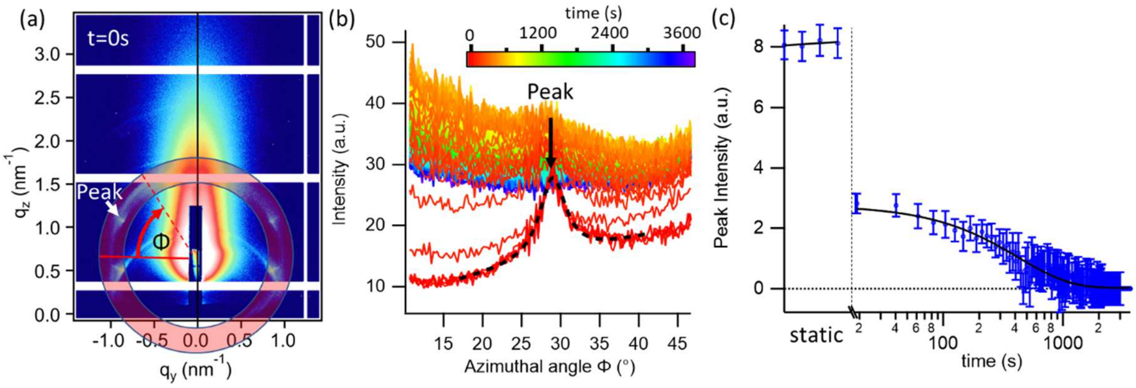

Figure S4: Azimuthal cut of GISAXS scattering pattern. Integration was done with specular reflected beam as beam center, as shown in (a). The azimuthal cut was done by integrating the 2D scattering pattern radially between 0.766 nm^{-1} inner radius to 1.132 nm^{-1} outer radius. The resulting

integrated intensity vs azimuthal angle Φ is shown in (b). Peak fitting is done with a Lorentzian, the peak intensity is shown in (c).

Transmission electron micrographs of free-standing Pt films

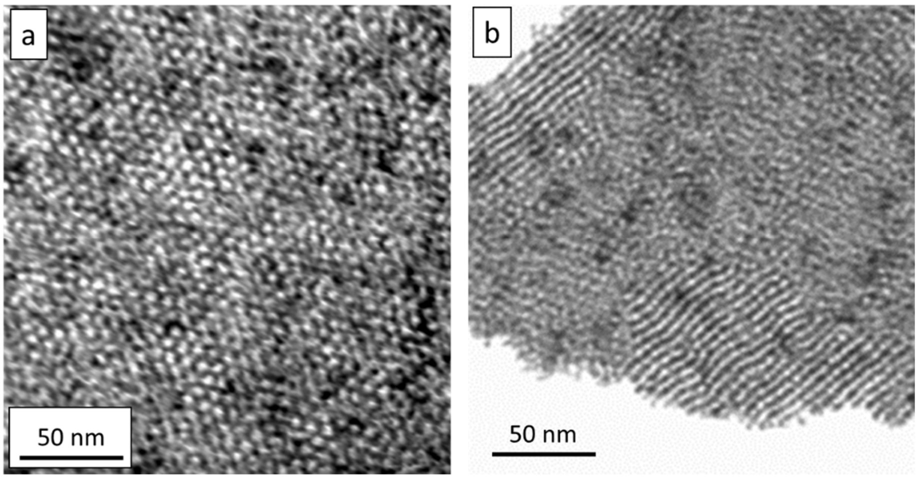

Figure S5: Transmission electron micrographs of a free-standing H_1-e Pt film. (a) Portion of the film with a largely regular hexagonal pore arrangement normal to the surface and (b) area showing also pore orientation parallel to the film surface.

SAXS measurement of Pt film in transmission

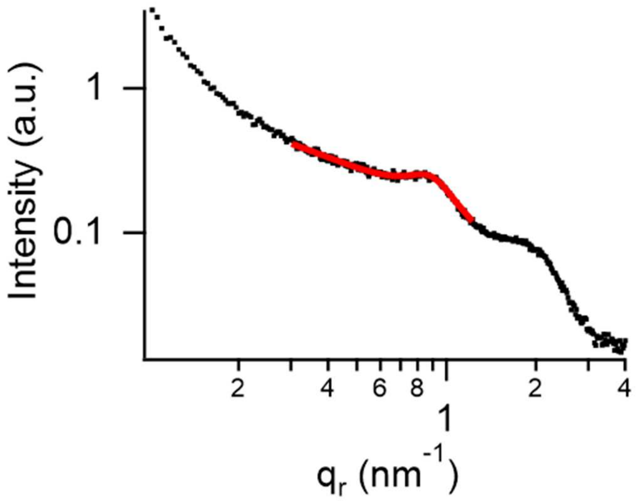

Figure S6: SAXS measurement in transmission of the mesoporous Pt film. We fitted the 1st order reflection with a Lorentzian peak and a power law background. The peak yields a d-spacing of 7.42(3) nm (q=0.847(3) nm^{-1}), in good agreement with the vertical streaks in GISAXS. The shoulder at ca. 2 nm^{-1} is attributed is attributed to the (11) and (20) reflections of the hexagonal phase and to the Pt morphology.

Cottrell fit of chronoamperometric data

Chronoamperometric data, i.e., current *I* vs time *t*, was fitted with the Cottrell equation, with coefficient *C*:

$$I = C\, t^{-\frac{1}{2}}$$

$$D = \frac{C^2\, \pi}{A^2\, c_0^2\, F^2\, n^2}$$

Data fitting yielded a diffusion coefficient D = 4.06E-08 cm^2/s, with bulk concentration $c_0 = 0.2044\frac{\text{mol}}{\text{l}} = 204.4\frac{\text{mol}}{\text{m}^3}$, geometrical surface area $A = 24\,.\,6\text{mm}^2 = 24.6 \times 10^{-6}\ \text{m}^2$, number of electrons involved in electrode reaction $n = 4$, Faraday constant $F = 96485$ C/mol. For determination of the goodness of fit, Pearson's chi-squared test yielded $\chi^2 = 2.2 \times 10^7$, χ^2_{reduced}=548.

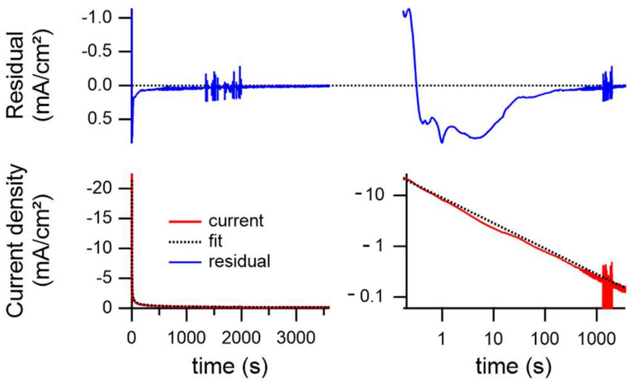

Figure S7: Current vs time during electrochemical deposition of mesoporous Pt. The double logarithmic plot shows linear behaviour, indicating diffusion-controlled behaviour of the current.

Power law fit of chronoamperometric data

Chronoamperometric data was fitted with a power law, with time t, background I_0, coefficient C_0 and exponent p:

$$I = I_0 + C_0 \cdot t^{\text{p}}$$

Data fitting yielded an exponent p = –0.52. For predicting the goodness of fit, Pearson's chi-squared test yielded χ^2 = 4.34x10^6, χ^2_{reduced} = 108.

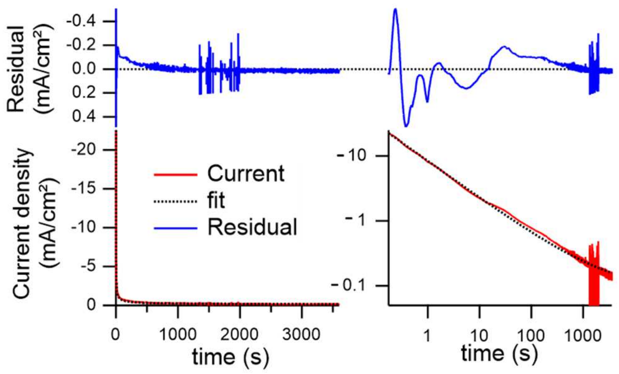

Figure S8: Chronoamperograms of Pt electrodeposition, fitted with power law.

Comparison of Current density deviation to normalized current density deviation

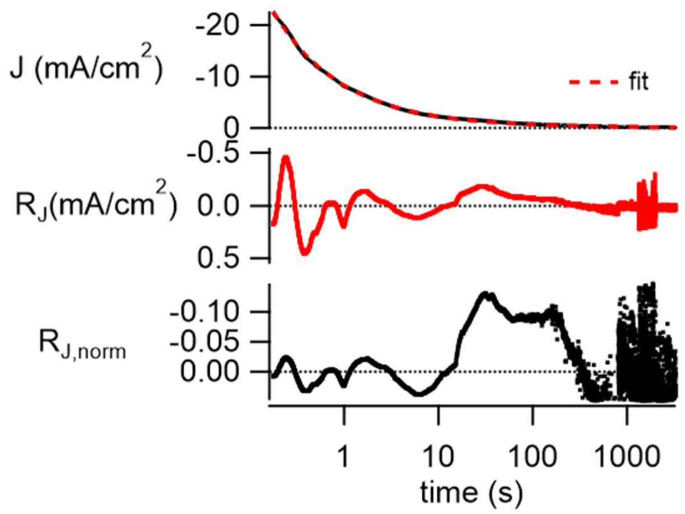

Figure S9: Current density J with the power law fit, shown in Figure 2b, are presented together with the fit residual R_J and the fit residual normalized by the current density $R_{J,norm}$. In $R_{J,norm}$, a broad peak between 15 and 350 s of electrodeposition is visible. Afterwards, the noise becomes dominant as the current density becomes lower.

In situ GISAXS pattern before and after electrodeposition, and ex situ after electrodeposition

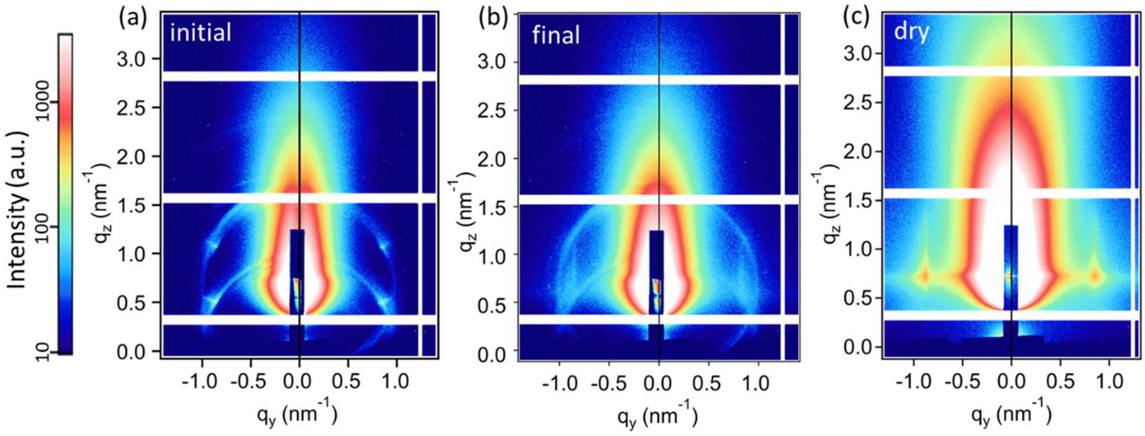

Figure S10: In situ GISAXS patterns (a) at initial stage and (b) at final stage, and (c) ex situ GISAXS pattern of the dry sample after electrodeposition and removal of electrolyte/template.

In operando GISAXS measurements without Pt salt

Electric double layer charging causing the loss of preferential orientation is excluded by CA measurements with the same potential, but the LLC electrolyte containing no Pt salt. The measurements showed no changes in intensity of the preferential orientation of the LLC, as shown in Figure S11.

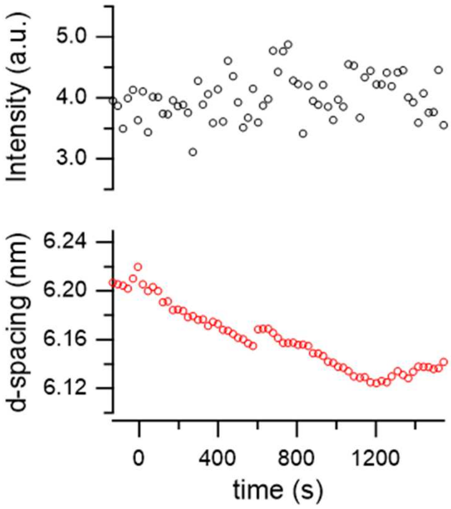

Figure S11: Intensity from the azimuthal cut performed while applying -0.069V vs Ag/AgCl to LLC template containing no Pt salt. SAXS analysis done as in Figure S4. No change in peak intensity visible, when chronoamperometry is started (-0.069 V vs Ag/AgCl, labelled start at time = 0 s), indicating that the orientation of the LLC in the electrolyte did not change. The decrease in *d*-spacing is associated with drying out of the electrolyte.

Estimation of film thickness at maximum pore ordering (at ca. 420 s)

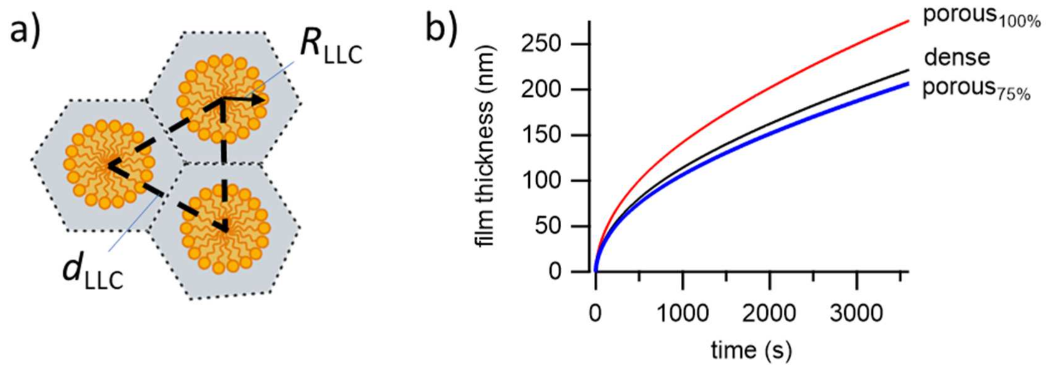

Figure S12: The thickness h of the electrodeposited Pt film was estimated from the deposition charge density σ_q, using Faraday's law. Assuming a dense non-porous Pt film, uniform thickness

and 100 % Coulombic efficiency, the thickness was calculated as $h = \sigma_q \frac{M}{z\,F\,\rho}$, with a molar mass M of Pt (195.084 g/Mol), valency z (4), Faraday constant F (9.6485 C/Mol) and mass density ρ (21.45 g/cm³)[1]. Furthermore, the LLC ordered pores were accounted for by introducing the volume fraction occupied by Pt f_{Pt}, so that thickness $h_{dense} = f_{Pt}\ h_{porous}$. The volume fraction f_{Pt} can be estimated from the cross section of the 2D hexagonal pore structure (a), as $f_{Pt} = \frac{A_{Pt}}{A_{all}}$, with $A_{Pt} = A_{all} - \frac{3}{6} R_{LLC}^2 \pi$ and $A_{all} = \frac{\sqrt{3}}{4} d_{LLC}^2$. Using radius $R_{LLC} = 3.2\ nm$, calculated from the form factor of the cylindrical micelles and the pore center-to-center distance d_{LLC} of the LLC (8.5 nm), an evolution of the thicknesses of the porous and the dense film can be estimated (b), assuming that the intermicellar spaces are completely filled with Pt and the micellar radius does not change during electrodeposition. From these calculations, at the maximum of the pore ordering (~420 s) the estimated film thickness was 92 nm (74 nm not accounting for the mesopores). As 92 nm were calculated assuming 100 % Coulombic efficiency, this value is to be taken as an upper limit. Assuming typical Coulombic efficiencies of ca. 75%[2], the porous film thickness is approximately 69 nm.

Fitting parameters at different positions

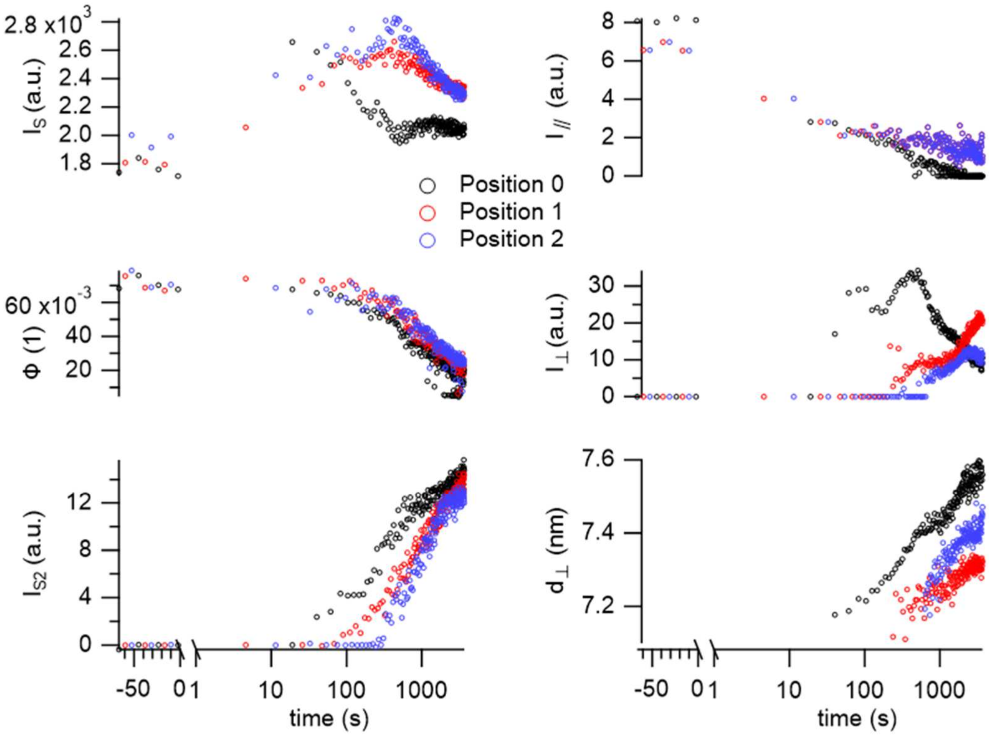

Figure S13: Fitting parameters of Schulz Sphere Parameters (left), and from vertical streaks. GISAXS measurements at three different positions. Subsequent steps I,II and III described in the main text, vary slightly depending on local deposition rates.

Contributions of the form factor of the LLC on the decrease in intensity $I_\perp$

Small changes in the *d*-spacing of the LLC can have a strong impact on the intensity of the XRD peaks if the peak position is close to a local minimum of the form factor (e.g., at $q \sim 1.2 nm^{-1}$ in Figure S14c). To estimate contributions of the form factor of the LLC on the decrease in intensity $I_\perp$ (Figure 4 in main text), the form factor was calculated from the small-angle x-ray diffraction pattern of the electrolyte (Figure S14a), using diffraction peaks (10),(11) and (20). The peaks were fitted with the Igor-inbuilt Multi-Peak Fit Package, using Gaussian distribution and a linear

background. A Lorentz correction was applied by multiplying each peak intensity by the square of the corresponding scattering vector, q_{hk}^2. The corrected intensities I_{hk} and scattering vectors q_{hk} were then fitted by model calculations interpreting the diffraction data with a simple two-phase model[3–5], decomposing the scattering electron density into aqueous domain and LLC with a cylindrical shape, respectively. Figure S14b shows the model, with density of ρ_{LLC} and a radius of R_{LLC} for the LLC domain and a density ρ_{aq} for the aqueous hexachloroplatinate domain. With this model, the corrected integrated intensities I_{hk} of the different diffraction peaks (hk) were fitted by applying $I_{\mathrm{hk}}(q_{\mathrm{hk}}) = K\left(\frac{2J_1(q_{\mathrm{hk}}R_{\mathrm{LLC}})}{q_{\mathrm{hk}}R_{\mathrm{LLC}}}\right)^2$, where K is a scaling constant (including the electron density contrast) and J_1 is the Bessel function of the first kind of first order.

Least-squares fitting yielded two values of radius R_{LLC} lower than halve of the d-spacing 6.5(3) nm of the LLC, namely 1.59(5) nm and 3.20(6) nm. Considering the size of the surfactant, an R_{LLC} of 3.20(6) nm makes more sense, as calculated from lamellar and hexagonal phases of mixtures of Brij and aqueous hexachloroplatinic acid solutions done by Asghar et al[6], which yielded an $R_{\mathrm{LLC,lamellar}}$ of 2.15(8) nm for the lamellar phase and 2.84(3) nm for the hexagonal phase ($R_{\mathrm{LLC,lamellar}} < R_{\mathrm{LLC,cylindrical}}$). The (10) reflection at q ~ 0.99 nm^{-1} (the only reflection visible in operando GISAXS) and the peak from the mesoporous Pt (q ~ 0.83 nm^{-1}) are at positions where the Form Factor has a negative slope. Therefore, a lower q (corresponding to higher d-spacing) would yield increased intensity.

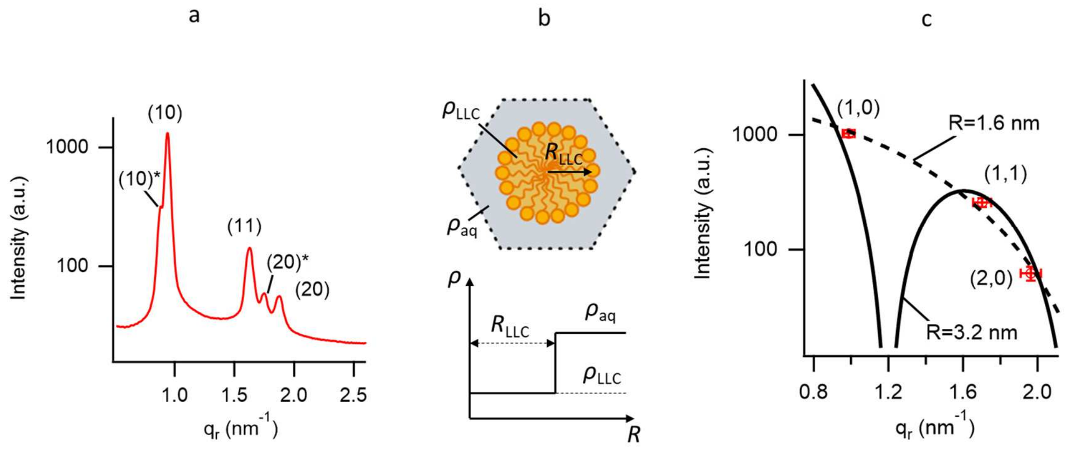

Figure S14: (a) SAXS pattern of electrolyte, with reflections (10),(11) and (20) as features of the hexagonal LLC. Reflections (10)* and (20)* derive from inhomogeneities in the hexagonal *d*-spacings. (b) two-phase model, with density of ρ_{LLC} and radius of R_{LLC} for the LLC domain, and density ρ_{aq} for the aqueous hexachloroplatinate domain. (c) Least-squares fit of the corrected intensities I_{hk} and scattering vectors q_{hk}, yielding two values of R, namely 1.59(2) nm and 3.20(3) nm. For both cases, the (10) reflection at $q \sim 0.99\ \mathrm{nm}^{-1}$ (the only reflection visible in operando GISAXS) and the peak from the mesoporous Pt ($q \sim 0.83\ \mathrm{nm}^{-1}$) are at positions where the Form Factor has a negative slope.

Scanning electron micrographs of Pt film

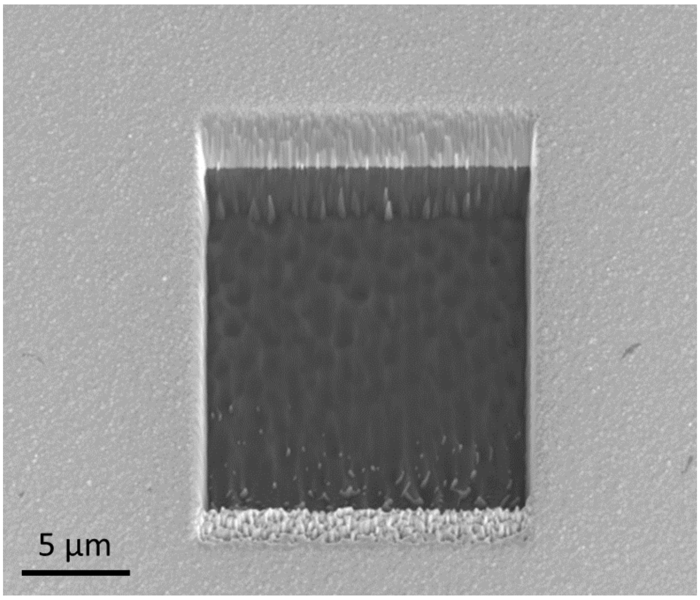

Figure S15: Scanning electron micrograph of H_1-e Pt film deposited on a gold-coated glass slide. To see the cross section, the sample was cut by focused ion beam milling.

References

(1) National Center for Biotechnology Information. *PubChem Compound Summary for CID 23939, Platinum*. PubChem Compound Summary for CID 23939, Platinum. https://pubchem.ncbi.nlm.nih.gov/compound/Platinum.

(2) Elliott, J. M.; Attard, G. S.; Bartlett, P. N.; Coleman, N. R. B.; Merckel, D. A. S.; Owen, J. R. Nanostructured Platinum (HI-EPt) Films: Effects of Electrodeposition Conditions on Film Properties. *Chem. Mater.* **1999**, *11* (12), 3602–3609. https://doi.org/10.1021/cm991077t.

(3) Imperor-Clerc, M.; Davidson, P.; Davidson, A. Existence of a Microporous Corona around

the Mesopores of Silica-Based SBA-15 Materials Templated by Triblock Copolymers. *J. Am. Chem. Soc.* **2000**, *122* (48), 11925–11933. https://doi.org/10.1021/ja002245h.

(4) Zickler, G. A.; Jähnert, S.; Wagermaier, W.; Funari, S. S.; Findenegg, G. H.; Paris, O. Physisorbed Films in Periodic Mesoporous Silica Studied by in Situ Synchrotron Small-Angle Diffraction. *Phys. Rev. B - Condens. Matter Mater. Phys.* **2006**, *73* (18), 1–10. https://doi.org/10.1103/PhysRevB.73.184109.

(5) Shyjumon, I.; Rappolt, M.; Sartori, B.; Cacho-Nerin, F.; Grenci, G.; Laggner, P.; Amenitsch, H. Mesostructured Silica Aerosol Particles: Comparison of Gas-Phase and Powder Deposit X-Ray Diffraction Data. *Langmuir* **2011**, *27* (9), 5542–5548. https://doi.org/10.1021/la104892s.

(6) Asghar, K. A.; Rowlands, D. A.; Elliott, J. M.; Squires, A. M. Predicting Sizes of Hexagonal and Gyroid Metal Nanostructures from Liquid Crystal Templating. *ACS Nano* **2015**, *9* (11), 10970–10978. https://doi.org/10.1021/acsnano.5b04176.
